# PTEN no Contexto da Revascularização do Miocárdio: A Ponta do Iceberg?

**DOI:** 10.36660/abc.20230170

**Published:** 2023-03-27

**Authors:** Leonardo Rufino Garcia, André Monti Garzesi, Marcello Laneza Felicio, Leonardo Antônio Mamede Zornoff

**Affiliations:** 1 UNESP Hospital das Clínicas de Botucatu e Faculdade de Medicina de Botucatu Serviço de Cirurgia Cardiovascular e Transplante Cardíaco São Paulo SP Brasil Serviço de Cirurgia Cardiovascular e Transplante Cardíaco – Hospital das Clínicas de Botucatu e Faculdade de Medicina de Botucatu – UNESP, São Paulo, SP – Brasil; 2 UNESP Faculdade de Medicina de Botucatu Departamento de Clínica Médica São Paulo SP Brasil Departamento de Clínica Médica - Faculdade de Medicina de Botucatu - UNESP, São Paulo, SP – Brasil

**Keywords:** Doenças Cardiovasculares, Doença da Artéria Coronariana/cirurgia, Revascularização do Miocardio/cirurgia, PTEN Fosfo-Hidrolase, Fosfatidilinositol-3 Kinase/metabolismo, Lipogênese, Glicólise, Apoptose, Proliferação de Celulas

As doenças cardiovasculares contribuem de forma significativa para a morbimortalidade no Brasil e no mundo. Dentro desse grupo de enfermidades, destaca-se a doença arterial coronariana, responsável por cerca de 12% dos óbitos totais no Brasil^1^ e é uma das principais causas de óbito na América do Norte.^2^

Além das mudanças do estilo de vida como abolição do tabagismo, perda de peso com prática regular de atividades físicas, controle de comorbidades, notadamente o diabetes e a hipertensão arterial sistêmica e o tratamento medicamentoso com antiagregantes plaquetários, betabloqueadores e estatinas, importante parcela dos pacientes necessita de tratamento objetivando a revascularização do miocárdio. Pacientes diabéticos, multiarteriais, com lesões no tronco da artéria coronária esquerda maiores do que 50% ou com função ventricular esquerda deprimida são os que mais se beneficiam.^3^ Esse fato é devido a diferentes fatores, incluindo diminuição da isquemia e dos sintomas, melhora da qualidade de vida, diminuição da ocorrência de infartos e, sobretudo, diminuição da mortalidade.^4^

A modalidade de revascularização melhor indicada para os pacientes com as características descritas acima é a cirúrgica, por apresentar vantagens como aumento das chances de revascularização completa, diminuição de futuras reintervenções coronarianas e menor chance de complicações quando comparada com intervenções percutâneas.^3^ Durante esses procedimentos, artérias e/ou veias autólogas são usadas para o by-pass de artérias coronárias parcial ou totalmente ocluídas por placas ateroscleróticas. As cirurgias podem ser feitas de acordo com uma variedade de técnicas, desde o uso ou não de circulação extracorpórea até procedimentos minimamente invasivos, utilizando apenas enxertos arteriais, sendo importante a escolha adequada da melhor estratégia para cada perfil de paciente.^3^

Apesar dos grandes avanços ao longo do tempo, como melhora da técnica cirúrgica propriamente dita e aumento da variedade dos tipos de procedimentos, avanços na anestesia e dos materiais utilizados, aceita-se que o uso de biomarcadores poderia adicionar informações prognósticas importantes. No entanto, pesquisas de caráter molecular são incipientes e pouco frequentes até o momento.^5^

Nesse sentido, destaca-se o estudo de Tahtasakal et al.,^5^ que teve como objetivo avaliar a expressão do gene PTEN e a expressão de sua proteína em amostras de tecido atrial direito e no sangue de 22 pacientes submetidos à cirurgia de revascularização do miocárdio em centro único. As amostras foram colhidas em dois momentos distintos, antes da cirurgia e após o término da circulação extracorpórea.

O gene PTEN (*phosphatase and tensin homolog deleted on chromosome 10*) localiza-se no cromossomo 10q23^6^ e foi inicialmente identificado em 1997^6,7^ como supressor tumoral.^7^ Estudos subsequentes classificaram-no também como regulador negativo de importantes vias de sinalização moleculares associadas com crescimento, proliferação e sobrevivência celulares, principalmente a via da fosfatidilinositol-3-kinase (PI3K)/AKT,^6^ concorrendo também para a modulação de outros processos biológicos, como lipogênese, glicólise e apoptose.^8,9^

O principal resultado apresentado pelos autores foi o aumento substancial tanto da expressão gênica quanto da proteína codificada pelo PTEN em amostras teciduais de átrio direito, reconhecido sítio biomarcador da proteômica cardíaca.^5^ Concluíram, então, sobre possível função do PTEN como marcador prognóstico de doença arterial coronariana nos próximos anos. Entretanto, alguns pontos ainda merecem discussão.

Primeiramente, todas as cirurgias foram realizadas com auxílio de circulação extracorpórea, reconhecido e seguro método que proporciona substituição das funções cardiopulmonares e facilita a confecção das anastomoses após o pinçamento aórtico e parada cardíaca diastólica. Devido, contudo, à passagem do sangue por superfícies não endotelizadas e ao fluxo sanguíneo não pulsátil, relevantes alterações podem ocorrer em maior ou menor intensidade na fisiologia. Na minoria dos casos são descritos síndrome da resposta inflamatória sistêmica, coagulopatias, síndrome do baixo débito cardíaco e lesão de isquemia- reperfusão miocárdica ao nível celular, por exemplo.^10^

Dessa forma, é importante esclarecermos sobre até que ponto o aumento da expressão gênica do PTEN e de sua respectiva proteína se deveu também ao estresse induzido pela circulação extracorpórea.

Outro aspecto a ser considerado é que outros fatores, como a hipóxia induzida pelo clampeamento aórtico e a reperfusão podem funcionar como fatores de estresse adicionais para os cardiomiócitos, que passam a obter grande parte de sua energia por meio da glicose, ao invés de ácidos graxos.^11^ Esse dado pode ter relação com aumentos da expressão do PTEN, que, como já dito, participa ativamente da regulação metabólica dos lipídeos e glicose e do metabolismo mitocondrial.^6^ Da mesma forma, é importante lembrarmos que o PTEN encontra-se sujeito a alterações pós- translacionais e a mecanismos regulatórios epigenéticos.^12^ Soma-se a esses dados o fato de outros genes atuarem em conjunto com o PTEN sobre a doença arterial coronariana^13^ em processos que ocorrem de forma simultânea.

Apesar das considerações acima e de ter avaliado apenas 22 pacientes, o estudo em questão tem alguns outros pontos que merecem destaque, como o de ser o primeiro a avaliar o gene PTEN no contexto da revascularização do miocárdio, aprofundando a análise subcelular durante procedimento extensivamente realizado e comprovadamente benéfico para vários subgrupos de pacientes. Dessa forma, abre- se caminho para novas pesquisas que objetivem a definição de marcadores prognósticos e novos alvos terapêuticos, nesse cenário clínico.
