# Supplementary material for: PTEN no Contexto da Revascularização do Miocárdio: A Ponta do Iceberg?
Source: Arq Bras Cardiol. 2023 Mar 27;120(4):e20230170. [Article in Portuguese] doi: 10.36660/abc.20230170 (PMC10263417; doi:10.36660/abc.20230170)
Supplement: Translation

# Translation: PTEN in the Context of Myocardial Revascularization: The Iceberg Tip?

**Authors:** Leonardo Rufino Garcia, André Monti Garzesi, Marcello Laneza Felicio, Leonardo Antônio Mamede Zornoff

**Affiliations:** 1 São Paulo SP Brazil Serviço de Cirurgia Cardiovascular e Transplante Cardíaco – Hospital das Clínicas de Botucatu e Faculdade de Medicina de Botucatu – UNESP, São Paulo, SP – Brazil; 2 São Paulo SP Brazil Departamento de Clínica Médica - Faculdade de Medicina de Botucatu - UNESP, São Paulo, SP – Brazil

**Keywords:** Cardiovascular Diseases, Coronary Artery Disease/surgery, Myocardial Revascularization/surgery, PTEN-Phosphohydrolase, Phosphatidyglinositol-3 Kinases/metabolism, Lipogenesis, Glycolysis, Apoptosis, Cells Proliferation

Cardiovascular diseases significantly contribute to morbidity and mortality in Brazil and worldwide. Within this group of diseases, coronary artery disease stands out, accounting for about 12% of total deaths in Brazil^1^ and is a leading cause of death in North America.^2^

In addition to lifestyle changes such as smoking cessation, weight loss with regular physical activity, control of comorbidities, notably diabetes and arterial hypertension, and drug treatment with antiplatelet agents, beta-blockers, and statins, a significant number of patients require treatment aimed at myocardial revascularization. Diabetic, multivessel-diseased patients with obstructions in the left main coronary artery greater than 50% or with depressed left ventricular function benefit the most.^3^ This fact is due to different factors, including a decrease in ischemia and symptoms, an improvement in quality of life, a decrease in the occurrence of infarcts, and, above all, mortality improvement.^4^

The best suitable revascularization modality for patients with the above characteristics is CABG (coronary artery bypass grafting surgery). It presents advantages such as increased chances of complete revascularization, reduction of future coronary artery interventions, and less chance of complications compared to percutaneous interventions.^3^ During these procedures, autologous arteries and/or veins bypass coronary arteries partially or completely occluded by atherosclerotic plaques. The surgeries can be performed according to various techniques, using or not cardiopulmonary bypass to minimally invasive procedures, with only arterial grafts. Indeed, the adequate choice of the best strategy for each patient profile is of utmost importance.^3^

Despite major advances over time, such as improvements in the surgical technique itself and an increase in the variety of procedures, advances in anesthesia, and the materials used, it is accepted that the use of biomarkers could add important prognostic information. However, research of a molecular nature is incipient and infrequent so far.^5^

In that regard, the study by Tahtasakal et al.^5^ is relevant. It aimed to evaluate the PTEN gene’s expression and its protein’s expression in right atrial tissue samples and the blood of 22 patients undergoing CABG in a single center. Samples were collected at two different times, before surgery and after the end of the cardiopulmonary bypass.

The PTEN gene (phosphatase and tensin homolog deleted on chromosome 10) is located on chromosome 10q23^6^ and was initially identified in 1997^6,7^ as a tumor supressor.^7^ Subsequent studies also classified it as a negative regulator of important molecular signaling pathways associated with cell growth, proliferation, and survival, mainly the phosphatidylinositol-3-kinase (PI3K)/AKT pathway^6^ contributing to the modulation of other biological processes, such as lipogenesis, glycolysis, and apoptosis.^8,9^

The main result presented by the authors was a substantial increase in gene expression and the protein encoded by PTEN in tissue samples from the right atrium, a recognized biomarker site of cardiac proteomics.^5^ They then concluded on the possible role of PTEN as a prognostic marker of coronary artery disease in the coming years. However, some points still deserve discussion.

First, all surgeries were performed under cardiopulmonary bypass, a recognized and safe method that replaces cardiopulmonary functions and facilitates the confection of anastomoses after aortic clamping and diastolic cardiac arrest. Due, however, to the passage of blood through non-endothelial surfaces and non-pulsatile blood flow, relevant changes may occur in greater or lesser intensity in physiology. In a minority of cases, systemic inflammatory response syndrome, coagulopathies, low cardiac output syndrome, and myocardial ischemia-reperfusion injury at the cellular level are described, for example.^10^

Thus, it is necessary to clarify the extent to which the increase in gene expression of PTEN and its respective protein was also due to the stress induced by cardiopulmonary bypass.

Another aspect to be considered is that other factors, such as hypoxia induced by aortic clamping and reperfusion, may act as additional stress factors for cardiomyocytes, which start to obtain a large part of their energy through glucose instead of fatty acids.^11^ This data may be related to increases in PTEN expression, which, as already mentioned, actively participates in the metabolic regulation of lipids and glucose and mitochondrial metabolism.^6^ Likewise, it is important to remember that PTEN is subject to post-translational changes and epigenetic regulatory mechanisms.^12^ Added to these data is that other genes act together with PTEN on coronary artery disease^13^ in processes that take place simultaneously.

Despite the above considerations and having evaluated only 22 patients, the study in question has some other points that deserve to be highlighted, such as being the first to evaluate the PTEN gene in the context of myocardial revascularization, deepening the subcellular analysis during an extensively performed procedure and proven to be beneficial for several subgroups of patients. Thus, the way is paved for new research that aims to define prognostic markers and new therapeutic targets in this clinical setting.
